# Anomalous Origin and Course of the Right Coronary Artery Misdiagnosed as Catheter-Induced Ascending Aortic Dissection

**DOI:** 10.1016/j.jaccas.2025.103282

**Published:** 2025-02-26

**Authors:** Martins Erglis, Uldis Strazdins, Sanda Jegere, Arnis Laduss, Andrejs Erglis

**Affiliations:** a Faculty of Medicine, University of Latvia, Riga, Latvia; b Latvia Center of Cardiology, Pauls Stradins Clinical University Hospital, Riga, Latvia

**Keywords:** coronary angiography, coronary artery bypass, coronary vessel anomaly, dissection, myocardial ischemia

## Abstract

A 55-year-old man was admitted for elective coronary angiography because of exertional chest pain. The angiogram revealed significant disease in the left anterior descending artery and the right coronary artery (RCA). Catheter-induced iatrogenic aortic dissection from the RCA ostium extending into the ascending aorta was suspected. During surgery, there were no signs of aortic dissection, but an anomalous origin of the RCA with an interarterial and intramural course was detected. The patient underwent a successful coronary artery bypass graft surgery.

## Graphical Abstract

**Figure:**
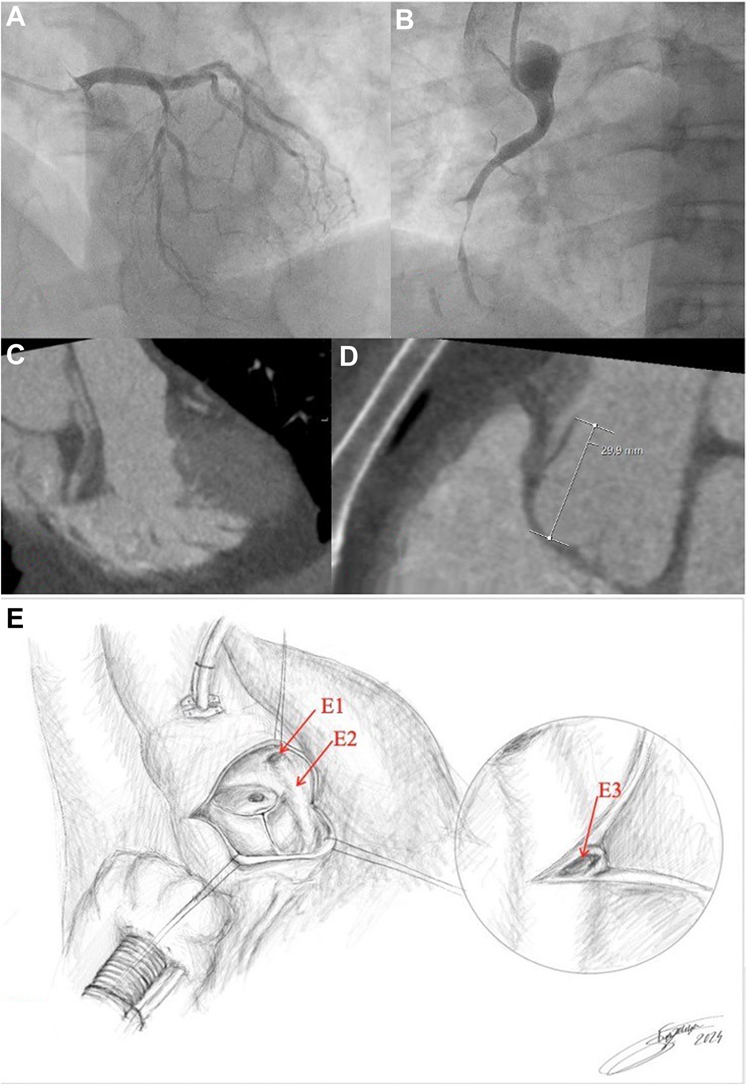

A 55-year-old man with a 6-month history of chest pain during moderate exercise was hospitalized for elective coronary angiography. Coronary angiography revealed 90% stenosis of the left anterior descending artery (Figure 1A, Videos 1 and 2). After multiple attempts to cannulate the right coronary artery (RCA), the ostium was discovered in the ascending aorta above the left main coronary artery. Angiography demonstrated subocclusion of the mid-RCA with iatrogenic dissection of the RCA extending into the ascending aorta (Figure 1B, Videos 3 and 4). Further investigation was discontinued because of the sudden onset of severe chest pain. Urgent computed tomography angiography revealed local dissection of the ascending aorta beginning at the RCA ostium, which the on-call radiologist interpreted as Stanford type A (Figures 1C and 1D). The heart team decided to perform emergent surgical repair of the acute Standford type A aortic dissection.

**Take-Home Message**

• A comprehensive diagnostic workup, which should include multimodal imaging with optimal image quality and interpretation as well as multidisciplinary collaboration, is essential for accurate diagnosis of rare coronary anomalies and for avoiding misdiagnosis.

**Figure 1:**
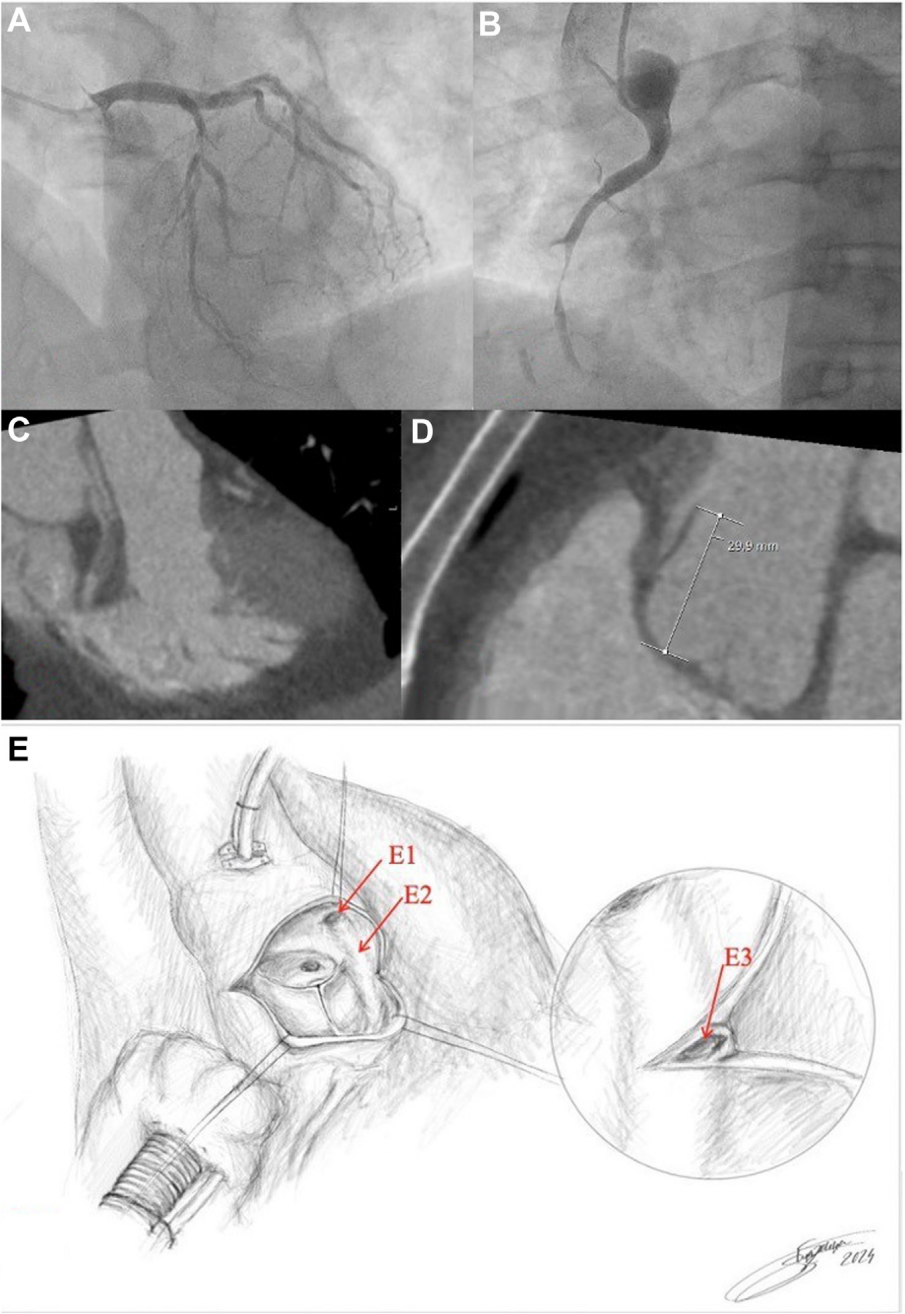
Imaging Assessment and Intraoperative Findings Coronary angiogram demonstrating a severe stenosis of the left anterior descending artery stenosis (A) and a subocclusion of the right coronary artery (RCA) and catheter-induced aortic dissection (B). (C and D) Coronary computed tomography angiography shows a dissection flap and signs of local dissection in the ascending aorta from the right coronary artery ostium (Stanford type A). (E) Schematic representation of the opened aortic root during the surgery shows an atypically localized ostium of the right coronary artery (E1) with the intramural course, illustrating the RCA's path within the aortic wall (E2). The lumen of the intramural RCA is exposed after the incision into the thickened aortic wall (E3). Illustration by Sintija Strazdina, 2024.

After median sternotomy and pericardiotomy, no macroscopic evidence of aortic dissection was found. Intraoperative transesophageal echocardiography did not demonstrate the dissection (Video 5). After initiation of cardiopulmonary bypass, the aorta was cross-clamped, and a transverse aortotomy was performed. Visual inspection and palpation of the aortic root did not reveal any signs consistent with aortic dissection. The left main coronary ostium was observed to be in its typical location within the left coronary sinus. However, the RCA ostium was not detected in the right coronary sinus. Approximately 30 mm above the aortic valve annulus, there was a funnel-shaped orifice measuring 9 × 5 mm within the left coronary sinus, which resembled a coronary artery ostium. Palpation disclosed intramural thickening extending from the orifice toward the aortic root. The aortotomy was extended to expose the thickened area, revealing a 5-mm-wide, half-moon-shaped arterial structure (Figure 1E). From the incision, an atraumatic probe was introduced. The probe path correlated with the typical anatomical location in the anterograde position and reached the orifice in a retrograde position. The incision into the RCA and aortotomy was closed. The patient underwent coronary artery bypass grafting where saphenous venous grafts were anastomosed to the RCA and first diagonal branch. The patient was discharged on postoperative day 8. At 1 year, the patient has remained asymptomatic.

We presented a case of anomalous origin and course of the RCA initially misdiagnosed as a retrograde extension of catheter-induced coronary artery dissection into the ascending aorta. Coronary artery anomalies are rare, affecting approximately 1% of the population.^1^ The management of this case posed several challenges. Computed tomography angiography suggested type A aortic dissection, prompting emergent surgical treatment. However, intraoperative exploration revealed the ectopic high aortic origin of the RCA with an interarterial and intramural course. In most cases, this anomaly is benign but poses a risk during cardiac surgery; therefore, surgeons should exercise caution to avoid cross-clamping or transecting the vessel.

This case report provides insights into potential diagnostic and treatment pitfalls caused by the anomalous origin and course of the RCA. It underscores the importance of considering rare coronary anomalies in patients presenting with chest pain, especially during elective procedures, and emphasizes the need for comprehensive diagnostic evaluation to guide appropriate therapeutic interventions.

## Case Summary

We presented a case of anomalous origin and course of the RCA initially misdiagnosed as a retrograde extension of catheter-induced coronary artery dissection into the ascending aorta. A 55-year-old man underwent an elective coronary angiography at our institution. During the investigation, significant 2-artery disease was noted, and catheter-induced iatrogenic aortic dissection from the RCA ostium extending into the ascending aorta was suspected. The emergent computed tomography angiography confirmed the diagnosis of Stanford type A acute aortic dissection. During surgery, there were no signs of aortic dissection, but an anomalous origin of the RCA with interarterial and intramural course was detected. This case report demonstrates that unfamiliar anatomical structures may sometimes be mistaken for pathologic conditions because of a lack of knowledge about these pathologies. As a result, the patient underwent surgery, although iatrogenic proximal coronary dissection extending into the aorta typically does not require emergency surgery, as conservative treatment is generally the standard approach. This case underscores the importance of considering rare coronary anomalies in patients presenting with chest pain, especially during elective procedures, and emphasizes the need for comprehensive diagnostic evaluation to guide appropriate therapeutic interventions.

## Funding Support and Author Disclosures

This study was funded by state research project VPP-EM-BIOMEDICĪNA-2022/1-0001, grants from the corporation “Sistēmu Inovācijas,” and the Latvian Innovative Medicine Foundation. The authors have reported that they have no relationships relevant to the contents of this paper to disclose.

## Appendix

Video 1 Coronary angiography of the left coronary artery shows a severely diseased left anterior descending artery with 90% stenosis (right anterior oblique, cranial view).

Video 2 Coronary angiography shows a severely diseased left anterior descending artery with 90% stenosis (left anterior oblique, cranial view).

Video 3 Coronary angiography of the right coronary artery shows a subocclusion of the middle part of the artery and catheter-induced dissection with contrast accumulation in the proximal part and above the ostium.

Video 4 Nonselective coronary angiography after a failed attempt to cannulate the right coronary artery shows contrast accumulation in the aortic wall.

Video 5 Intraoperative transesophageal echocardiography in the long-axis view shows a possible dissection membrane. However, when color Doppler was applied, the laminar flow without turbulence was noted, disapproving the initial diagnosis of aortic dissection.
